# Realization of Low Profile Leaky Wave Antennas Using the Bending Technique for Frequency Scanning and Sensor Applications

**DOI:** 10.3390/s19102265

**Published:** 2019-05-16

**Authors:** Abhishek Kandwal, Zedong Nie, Lei Wang, Louis W. Y. Liu, Ranjan Das

**Affiliations:** 1 Shenzhen Institutes of Advanced Technology, Chinese Academy of Sciences, Shenzhen 518055, China; abhishek@siat.ac.cn (A.K.); wang.lei@siat.ac.cn (L.W.); 2 EEIT Department, Vietnamese German University, Binh Duong 820000, Vietnam; liu.waiyip@vgu.edu.vn; 3 Indian Institute of Technology (IIT), Bombay 400076, India; ranjandas@ee.iitb.ac.in

**Keywords:** transmission line, modes, leaky wave, scanning, dispersion, modulation

## Abstract

This paper proposes an efficient transmission line modulation by using the bending technique to realize low profile leaky wave antennas in the Ku-band for frequency scanning and sensor applications. The paper focuses mainly on the bending effects of the transmission line in terms of the sharpness of edges. The right-hand/left-hand transmission line can be designed in the form of zig-zag pattern with sharp corners and only the right-hand transmission line in the form of sinusoidal patterns with smooth corners. In this presentation, we demonstrate that transmission lines of this kind can be used to realize highly efficient leaky wave antennas with broadband impedance matching and high gain characteristics in the Ku-band. Dispersion analysis and ladder network analysis have been performed for investigating the performance of the proposed designs. The sharpness of the bends periodically distributed along the body of the antenna has been used to our advantage for frequency scanning in the left-hand and right-hand quadrants at different frequencies. The proposed bending technique has been proven to be instrumental in achieving the desired characteristics of low profile leaky wave antennas.

## 1. Introduction

Beaming electromagnetic waves is highly important in communication applications and in the formation of high contrast images in medical imaging. Beaming an electromagnetic wave with high gain at multi-band frequencies normally requires a two-dimensional antenna array. At the time of writing, the majority of two-dimensional antenna arrays are embedded with a complex feeding network that simultaneously excites each antenna element, but this mechanism inevitably incurs significant losses, especially at high frequencies. In addition, two-dimensional antenna arrays are extremely bulky. There is an obvious demand for antennas with a low profile, high directivity, and the feasibility of scanning at multi-band frequencies. In this paper, we propose a methodology for designing a small-sized dual-band or multiple band antenna, which in essence overcomes the above problems, and due to its compactness, it can be used for many applications in the frequency of operation [1,2,3,4,5,6].

One of the most unique features of left-hand materials is their negative permittivity and permeability. Over the past few decades, left-handed materials have been highly popular in the research communities because of their unprecedented characteristics. Composite right-hand/left-hand (CRLH) transmission lines have been used in the implementation of leaky wave antennas with the ability to perform frequency scanning of an electromagnetic beam from backfire to endfire. As of right now, however, most of the antennas falling into this category contain vias and interdigital structures, which not only increase the degree of design complexity, but also limit their applications. In recent years, most research groups have been focusing on novel designs that increase the pattern bandwidth of the antenna and that reduce the thickness of the antenna [7,8,9,10,11,12,13,14].

The emergence of dual-band or multi-band leaky wave antennas (LWA) with CRLH structures and dual CRLH structures for beam scanning applications has enormously advanced the field of leaky wave antennas [15,16]. Substrate integrated waveguide (SIW)-based leaky wave antennas on one the one hand are simple to design to achieve leaky wave characteristics, but on the other hand, they increase the cost. SIW leaky wave antennas leak out the energy through their holes or slots. These types of antenna can steer the beam in a forward and backward direction, which also offers dual-band operations. Recently, an SIW based on CRLH structures has been used to achieve a three-band substrate integrated waveguide leaky wave antenna. These antennas may contain two CRLH bands along with a new right-handed (RH) band in between. In our previous work [17], a modified lossy transmission line model has already been developed to analyze and to synthesize non-uniform leaky wave antennas where a forward and reverse frequency scanning has been achieved in the right-hand quadrant, but not in the left-hand region.

With the help of a truncated superstrate, another leaky wave-based dual-band phased array has been achieved for on-board satellite communications with the bandwidth extended for use in the K-band (17.7–20.2 GHz) and the Ka-band (27.5–30 GHz) [17]. Some leaky wave antenna designs have been proposed recently for Ku-band applications, but are mostly based on substrate integrated waveguides. These designs have achieved good antenna performance, but at the expense of increased fabrication costs and design complexity. Furthermore, this is to point out that very limited research has been done on microstrip leaky wave antennas in the Ku-frequency band [15,16,17,18,19,20,21,22,23,24,25,26]. In this work, the sharpness of the bends along the body of a microstrip transmission line is advantageously used to realize a fundamentally different leaky wave antenna with a broadband impedance matching and high gain characteristics in the Ku-band. Our attention has been channeled to cost-effectively increase the overall antenna efficiency with a widened bandwidth, with lower weight, and with a reduction in fabrication costs [4,27,28]. The main objective of the paper is to propose microstrip LWAs with a low profile and to analyze the bending effects. The bending effects in a microstrip transmission line need to be analyzed rigorously along with the dispersion analysis. The authors have made an effort to propose designs that are low profile, compact, easy to fabricate, low cost, and have achieved good antenna characteristics with frequency scanning in the operating frequency band.

## 2. Concept of Periodically-Distributed Bends Along a Microstrip Transmission Line

Leaky wave antennas normally radiate from the slots and edges of the structures. These slots and edges will lead to radiation of energy in some particular directions according to the designs. Transmission lines periodically loaded with bends with different sharpness tend to radiate differently due to various perturbations. Conventional transmission lines on the other hand can only propagate waves in one direction and cannot radiate on their own unless they have some perturbations or modulations. The whole idea is to turn these conventional transmission lines into periodically-loaded transmission lines to achieve leaky wave radiation.

In the proposed antenna, a homogeneous transmission line with multiple periodically-loaded sharply-discontinuous sections is employed for the excitation of leaky waves on the broadside with the help of its intrinsic negative permittivity and permeability. The idea of this antenna was originally inspired by the observations from our previous work, which did not support the left-hand features: (1) the observation that CRLH behavior was found in sharp bends of a modified transmission line; (2) right-hand quadrant scanning was found to be possible with a smooth edge sine wave modulated transmission line; and (3) the generated CRLH behavior can be modified to facilitate backward and forward scanning.

**Figure 1:**
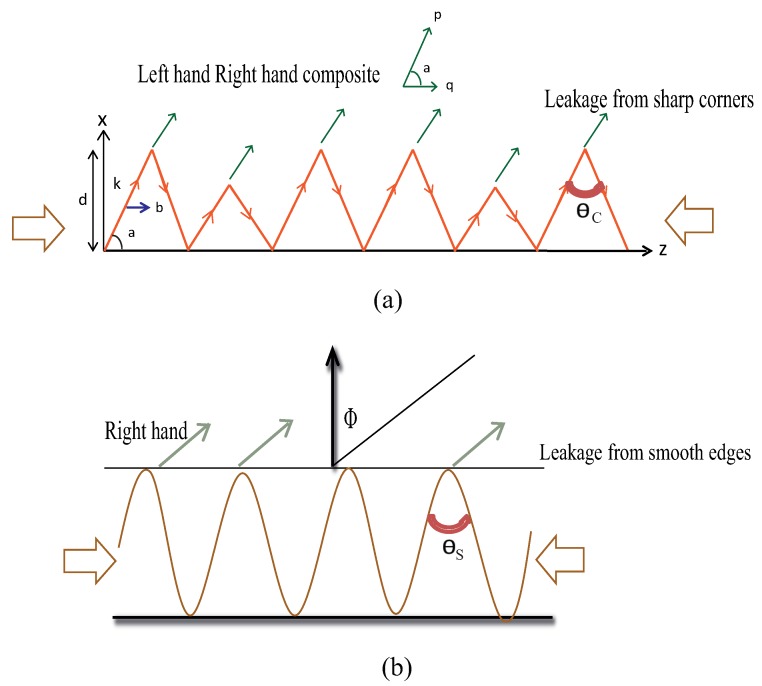
Principle of the proposed designs: (**a**) sharp edge (**b**) smooth edge.

Along the body of the antenna, leakage of superimposed electromagnetic waves fed by a single input port boosts the overall antenna gain even though the proposed antenna has a low profile in the direction of radiation. The proposed LWAs supports the propagation of a traveling wave, which continuously radiates through the sharp discontinuities along the body of the antenna so as to generate a highly-focused beam of radiation. The overall topology of the proposed antenna eliminates the need for conventional signal feeds with added phase shifters or complicated signal feeding lines, which are not only high profile, but also technically complicated. As a whole, low profile leaky wave antennas for Ku-band applications, frequency scanning applications, as well as sensor networks have been implemented. An efficient transmission line modulated with bends of different sharpness has been realized and analyzed using model analysis. Furthermore, CRLH behavior has been observed in a transmission line modulated with sharp bends, which features backward and forward scanning (see Figure 1). On the other hand, right-hand quadrant scanning has been observed in a transmission line periodically loaded with a smooth edge sine wave pattern (see Figure 1).

Efficient, high gain, and broadband characteristics have been achieved by simply introducing bends in the transmission line without introducing any stubs or components or any other SIW structures, which are comparatively more typical and costly. It has further been proposed that transmission lines periodically loaded with sharp bends or smooth bends have completely different impacts on radiation and frequency scanning. In our model analysis, a unit cell with sharp bends provides frequency scanning in both directions, i.e., forward and backward quadrants along with a dual-frequency operation. Furthermore, another unit cell with a smooth corner has been proposed for changing the radiation direction and scanning in the forward quadrant.

The main point is that different kinds of frequency scanning features have been observed when the transmission line was periodically loaded with sharp or smooth bends at a particular angle. The angle at which each unit cell of the transmission line has been bent further decides the direction of propagation of the wave and hence the direction of radiation from the bends. After rigorous optimization, it has been observed that high gain and better radiation properties have been obtained at a bending angle of 56 degrees for the proposed designs. For the sharp edge design, when the angle of the bend was reduced from 56 degrees (<56 deg.), it changed the cutoff frequencies. The cutoff frequency went higher in this case. Further, it significantly changed the radiation as the gain reduced with the increase in the beamwidths. When the angle was increased from 56 degrees (>56 deg.), again, a similar kind of phenomenon has been observed and degraded the radiation performance. In the case of smooth edge sine wave design, when the angle of the bend was reduced from 56 degrees (<56 deg.), the operating frequency region modified. The higher cutoff frequency for Mode-2 has been observed at 15 GHz. Further, there was no mode coupling at the 60-degree phase, which is one of the reasons for the modified operating frequency bands. When the angle was >56 degrees, the mode coupling was significantly less, and it occurred at the 120-degree phase. For both cases, broad beamwidths have been observed, which reduced the overall gain.

The proposed principle, which was originally inspired by our analysis on a lattice model, has been validated with the dispersion analysis, the results of which have validated the measured propagation performance and radiation characteristics of the proposed right-hand/left-hand transmission line design with sharp corners and the right-hand transmission line design with smooth corners.

## 3. Proposed Ku-Band Sharp Bent Right-Hand/Left-Hand Transmission Line-Based LWA

### 3.1. Dispersion Analysis

**Figure 2:**
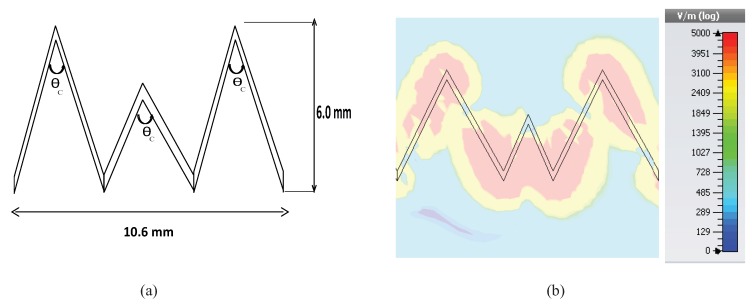
(**a**) Unit element of the proposed LWA antenna and (**b**) field distribution.

The proposed unit cells with a sharp bend have been analyzed to study the dispersion behavior of different modes. Figure 2 shows the geometry of the unit element and its field distribution. As can be seen from the field distribution, the radiation was mostly leaking out from the edges. The sharply-bent unit cell on a thin Rogers Duroid 5880 substrate has been simulated using the electromagnetic eigen mode solver CST. The substrate has a thickness of 0.5 mm and a dielectric constant of 2.2. The period of the unit element/cell was 10.6 mm in the horizontal x-direction and 6 mm in length towards the vertical y-direction.

**Figure 3:**
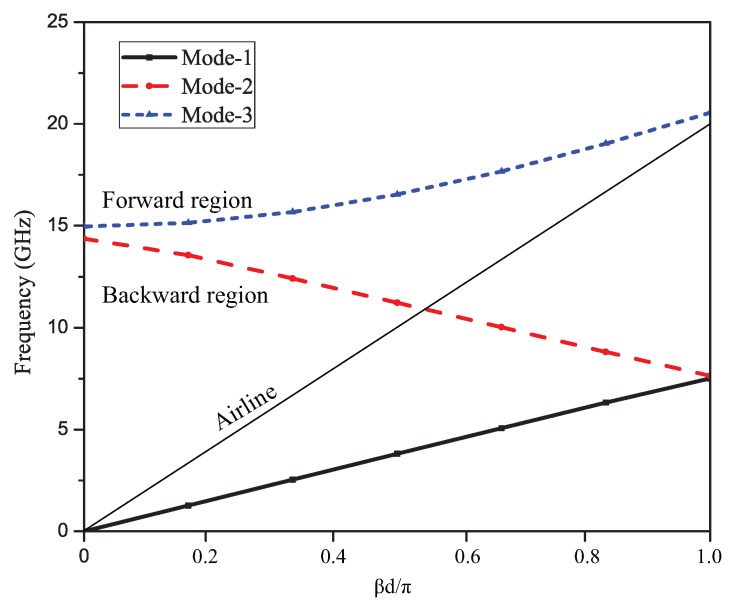
Dispersion curve for unit element: The slow wave and fast wave regions separated by an air line.

The angle of each bend in each of the unit cells was 56 degrees. The dispersion graph showing different modes of the proposed unit element is depicted in Figure 3. The dispersion analysis presents three modes viz. the dominant mode as quasi-TEMmode, the first higher order mode, and the second higher order mode. The slow wave modes and fast wave modes have been separated by the air line, which served as a reference for the dispersion analysis. The slow wave mode was the quasi-TEM mode, which was below the air line, as can be seen in the figure, and is termed as a slow wave mode due to its lower phase velocity than air. The fast wave modes are shown in the graph above the air line, which had a phase velocity higher than the speed of light. The fast wave modes were generally responsible for scanning features. The air line will divide the dispersion graph into the region of operation in the left-hand backward quadrant and in the right-hand forward quadrant. As shown in Figure 3, at frequencies below 14.5 GHz, the antenna exhibited a left-hand quadrant behavior and, at frequencies above 15 GHz, the antenna exhibited a right-hand quadrant behavior. Furthermore, the upper and lower frequency limits can be defined by this dispersion diagram. This analysis can be further verified by analyzing the reflection coefficient graph, i.e., S-parameter graph.

**Figure 4:**
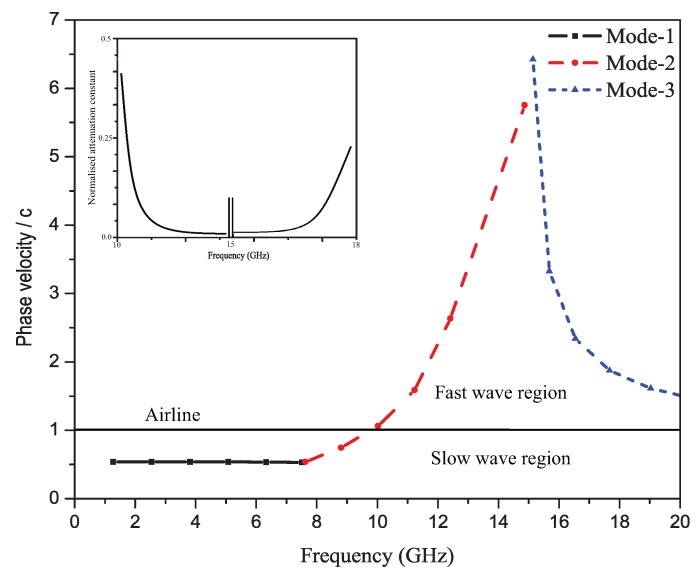
Phase velocity plot of the unit cell (inverse propagation constant) and attenuation constant (leakage).

Figure 4 depicts the propagation constant graph that shows the variation of the phase velocity (Vp) with the frequency and curve for the normalized attenuation constant. Furthermore, the curve plotted is Vp/cas a function of frequency, where ’c’ is the speed of light. This graph shows the inverse normalized propagation constant. The frequency region below the air line (see Reference Point 1) is the slow wave region and above the air line is the fast wave region. This graph further verifies the frequency of operation of the proposed unit element in fast wave region. The left-hand frequency region shows the backward scanning and the right-hand frequency region the forward scanning.

### 3.2. Validation and Implementation of Proposed Ku-Band LWA

**Figure 5:**
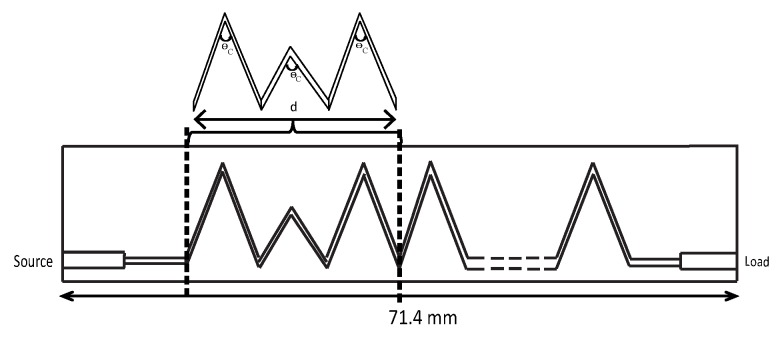
Geometry of the proposed Ku-band leaky wave antenna (length = 71.4 mm, d = period of each unit element, $\theta_{C}$ = bending angle.)

**Figure 6:**
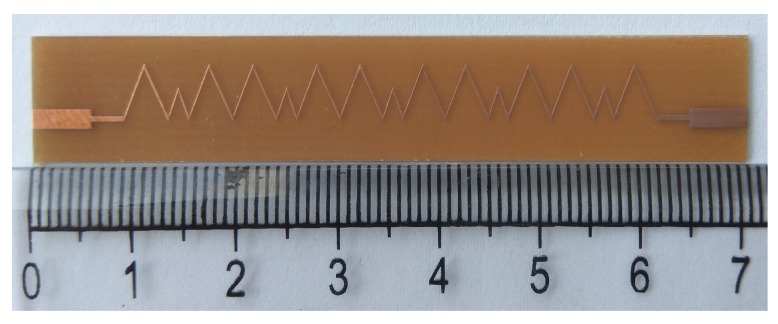
Prototype of the proposed Ku-band leaky wave antenna.

Figure 5 shows the geometry of the proposed Ku-band leaky wave antenna. The length of the antenna was 71.4 mm with the period “d” of each unit element equal to 10.6 mm. The proposed antenna has been fabricated using the standard fabrication technology. The dielectric constant of the substrate was 2.2, and it had a 0.5-mm thickness. The fabricated prototype of the proposed dual band leaky wave antenna is shown in Figure 6. For simulating the design, the transient solver in CST Studio Suite has been used.

**Figure 7:**
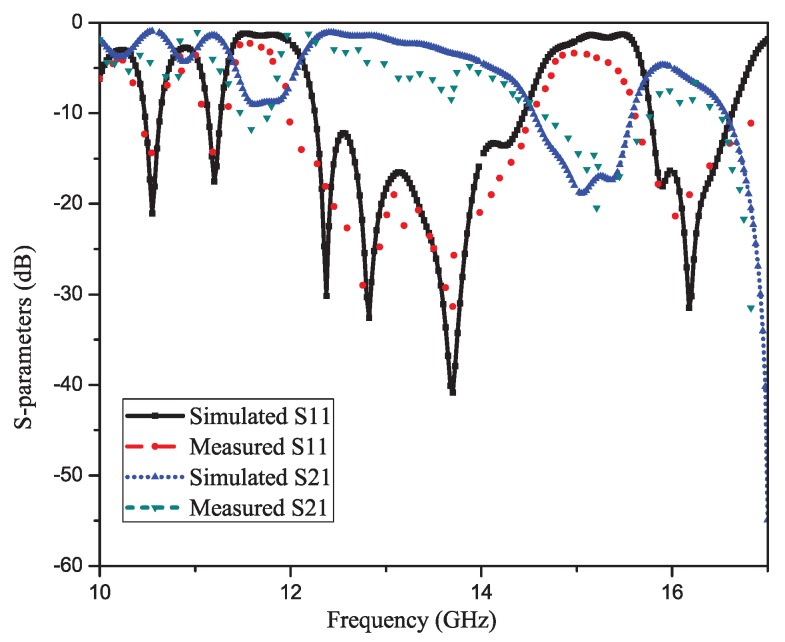
S-parameters of the proposed Ku-band leaky wave antenna (reflection S11 and transmission S21 parameters: simulated and measured).

Figure 7 depicts the simulated and measured results of the S-parameter (S${}_{11}$ and S${}_{22}$) for the proposed antenna design. It can be clearly seen from Figure 7 that the antenna has achieved a broadband impedance matching in the frequency region of the Ku-band. The maximum return loss was around 40 dB. S${}_{21}$ varied from −5 dB–−10 dB across the whole frequency band. This shows a very good leakage property of the proposed leaky wave antenna. A dual resonance has been obtained in the Ku-band as desired and estimated.

**Figure 8:**
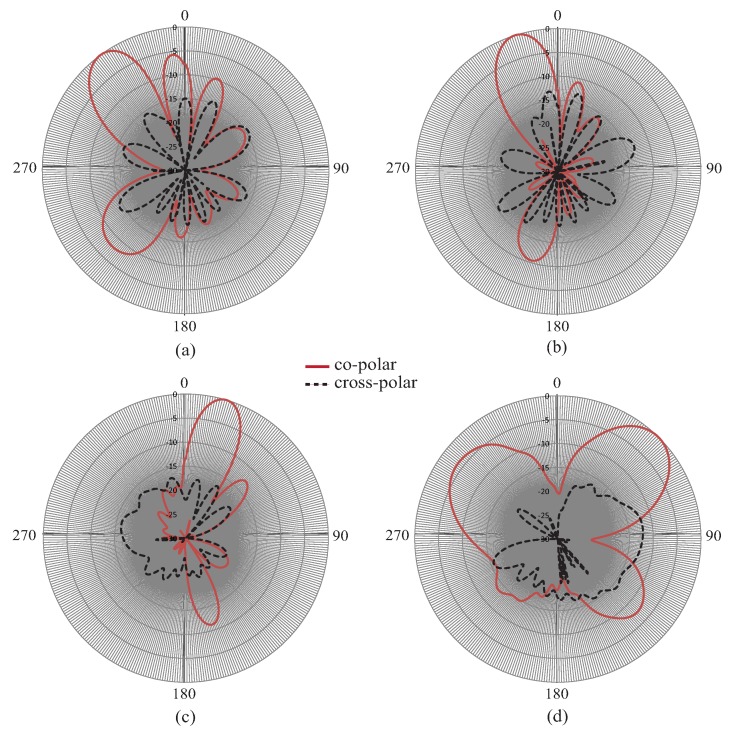
Measured radiation patterns (co-polar and cross-polar) with scanning: (**a**) 13 GHz, (**b**) 14 GHz, (**c**) 16 GHz, (**d**) 16.5 GHz.

Figure 8 shows the measured co-polar and cross-polar radiation patterns of the proposed Ku-band CRLH leaky wave antenna. Figure 8a is the pattern at 13.0 GHz, Figure 8b the pattern at 14.0 GHz, Figure 8c the pattern at 16.0 GHz, and Figure 8d the pattern at 16.5 GHz. It can be observed from the results that the antenna has achieved forward scanning at frequencies of 16.0 GHz and 16.5 GHz and backward scanning at frequencies of 13.0 GHz–14.0 GHz. This type of left-hand right-hand scanning was obtained as a result of the fast wave mode defined by the air line in the dispersion diagram. The gain obtained was very good throughout the frequency bands of operation varying from 11 dBi–14 dBi. At a frequency of 13.0 GHz, the main beam direction was about −30 degrees and at 17 GHz, the main beam scanned to +45 degrees. This phenomenon has already been discussed in the sections above and was a result of the CRLH-type behavior of higher modes. The results of this investigation have proven beyond any doubt that forward and backward scanning has been observed in the two frequency bands of operation. The minimum cross-polar levels obtained were around −18 dB in the operating frequency band.

**Figure 9:**
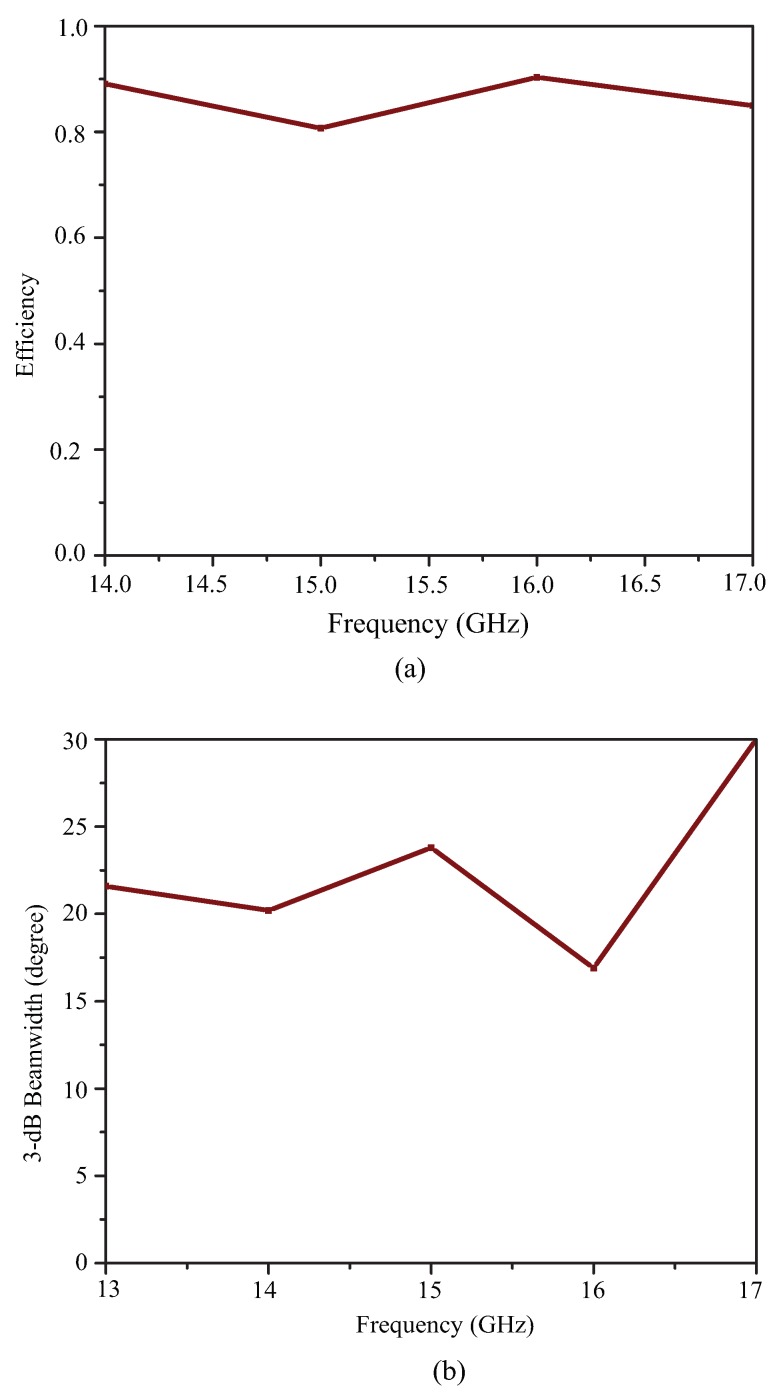
(**a**) Efficiency of the proposed Ku-band leaky wave antenna and (**b**) 3-dB beamwidth variation with frequency.

Figure 9a depicts the efficiency and Figure 9b depicts the 3-dB beamwidth graph of the proposed leaky wave antenna as a function of frequency. The efficiency has improved from 80–95 percent as the frequency increased from 14.0 GHz–17.0 GHz. Due to the increase in the leakage radiation, the gain increased from lower frequencies to higher frequencies. At higher frequencies, the efficiency reached 90–95 percent, satisfying the criteria for leaky wave generation. Figure 9b shows the variation of the 3-dB beamwidth with the frequency. The beamwidth was slightly higher due to the fact that the attenuation constant may be large at a few frequency points especially at the extreme ends of the frequency band.

**Figure 10:**
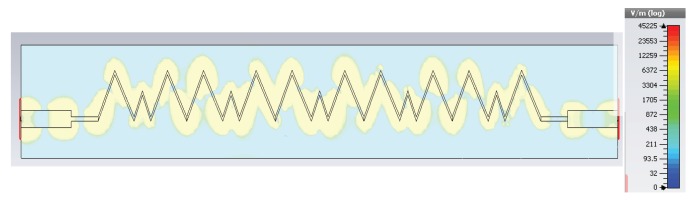
Field distribution of the proposed Ku-band leaky wave antenna.

**Figure 11:**
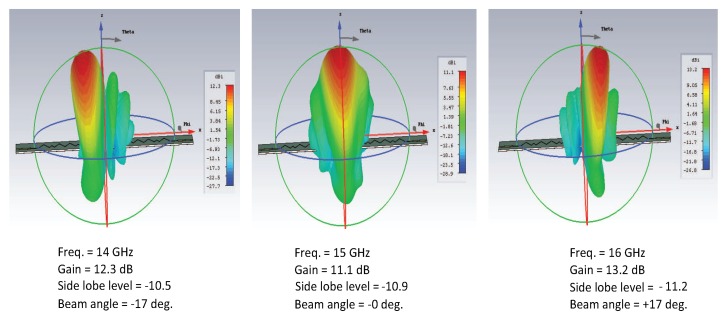
3D radiation patterns showing forward and backward scan.

Figure 10 shows the E-field distribution, and Figure 11 shows the 3D radiation patterns at different frequencies for the proposed leaky wave antenna. The 3D pattern has clearly shown the scanning feature and beam directions at different frequencies in the left-hand and right-hand quadrants.

## 4. Proposed Right-Hand Sine Wave Smooth Edge Transmission Line-Based LWA

### 4.1. Dispersion Analysis

**Figure 12:**
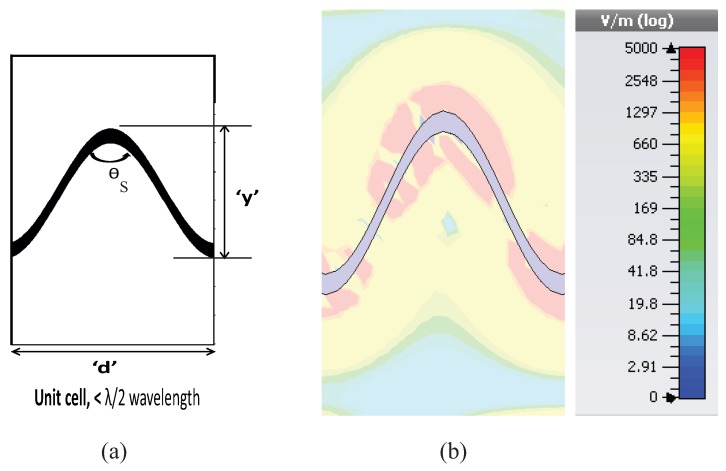
(**a**) Unit cell of the sine wave LWA and (**b**) field distribution.

The transmission line periodically loaded with a smooth edge sine wave unit cell exhibited unidirectional-scanning characteristics, i.e., only right-hand radiation. The unit cell has been extracted to perform the dispersion analysis. The geometry of the single unit element is plotted in Figure 12. Figure 12 also shows the field distribution pattern of the unit element where most of the radiation leaks out of the smooth bent corner. CST simulation has been done for the smooth-edge sine wave modulated unit element. The dimensions of the element were as follows: the period of the unit element/cell “d” was equal to 6.0 mm in the x-direction, and its height was equal to 5.0 mm in the y-direction. The angle of each bend in the unit cell was 56 degrees.

**Figure 13:**
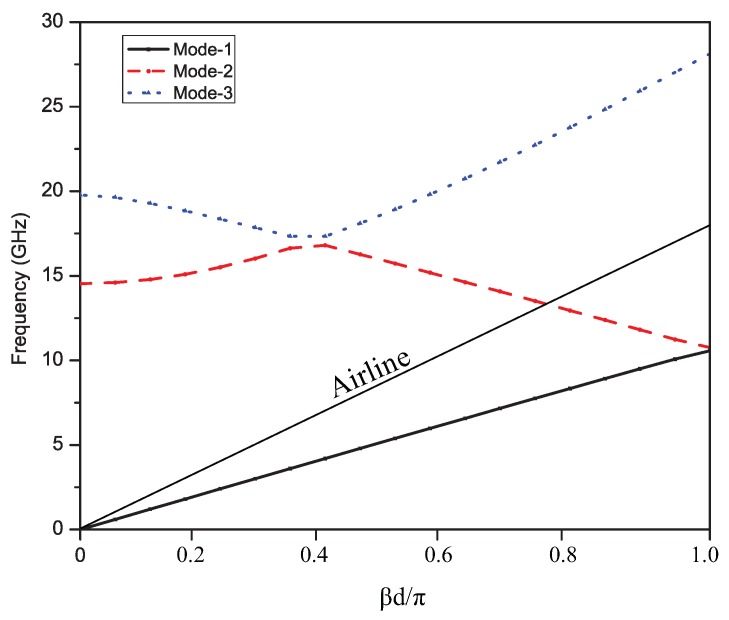
Dispersion curve of the sine wave LWA: the slow wave and fast wave regions separated by the air line.

**Figure 14:**
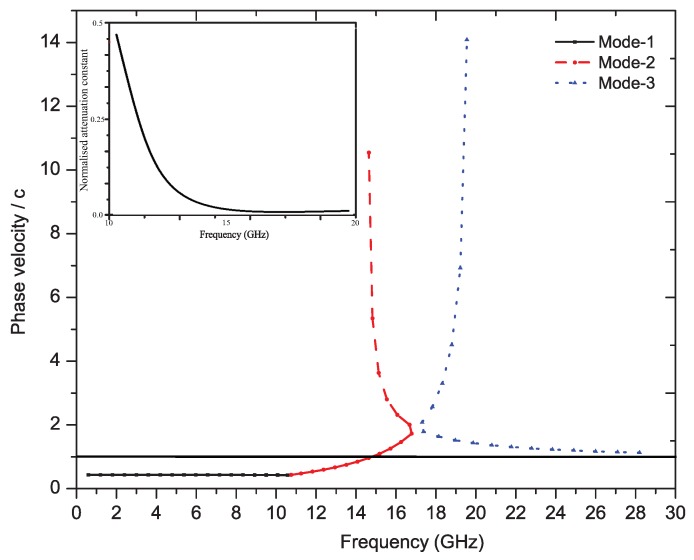
Phase velocity of the sine wave LWA (inverse propagation constant) and attenuation constant (leakage).

Figure 13 shows the dispersion diagram of the proposed unit element. As before, the dispersion curve presents three modes, i.e., dominant mode, the first higher order mode, and the second higher order mode. The slow wave modes and fast wave modes have been separated by the air line, which served as the reference. The slow wave mode was the quasi-TEM mode, which was below the air line and had a phase velocity less than the speed of light. The fast wave modes are shown in the graph above the air line, which had a phase velocity higher than the speed of light. The fast wave modes were in general responsible for the desired scanning feature. Furthermore, the upper and lower frequency limits were defined by this dispersion diagram. This analysis can be further verified with the help of the S-parameter graph, which will be discussed in a later section of this paper. Figure 14 shows the normalized propagation constant and normalized attenuation constant curve. The curve plotted is intended to show the inverse normalized propagation constant, i.e., Vp/c as a function of frequency. This graph has verified the frequency of operation of the proposed unit element in the fast wave region, i.e., the right-hand quadrant. This unit element had smooth bends with the same angle, i.e., 56 degrees. By comparing both shapes, it can be concluded that the sharpness of the bends had a great impact on the antenna behavior, especially in terms of the radiation characteristics.

### 4.2. Implementation and Results of the Proposed Right-Hand Sine Wave LWA

This section describes the implementation of the proposed sine wave leaky wave antenna. The antenna properties obtained viz. antenna reflection-transmissions, radiation, leakage, and efficiency have been validated with the help of measurements. As explained previously, the smooth bend(s) in the unit element will result in the frequency scanning in the right-hand quadrant.

**Figure 15:**
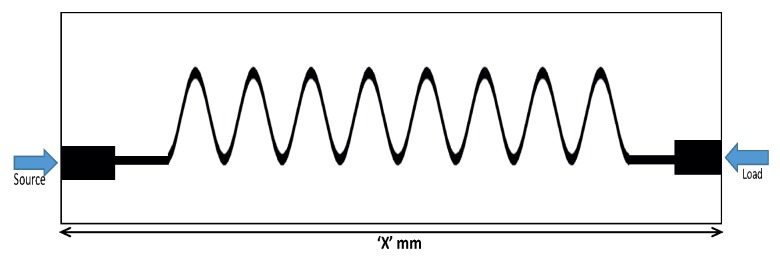
Geometry of the proposed sine wave leaky wave antenna.

**Figure 16:**
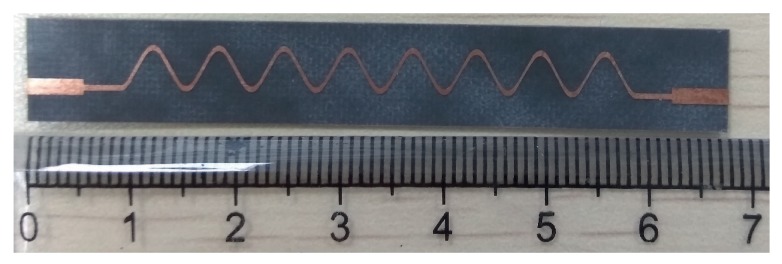
Prototype of the proposed sine wave leaky wave antenna.

Figure 15 shows the geometry of the proposed smooth-edge sine wave leaky wave antenna. The length of the antenna was 68.2 mm. The period of each unit cell was 6.0 mm. The proposed antenna was fabricated on a Rogers Duroid 5880 substrate using the standard fabrication technology. The dielectric constant and the thickness of the substrate were respectively 2.2 and 0.5 mm. The fabricated prototype of the proposed sine modulated leaky wave antenna is shown in Figure 16.

**Figure 17:**
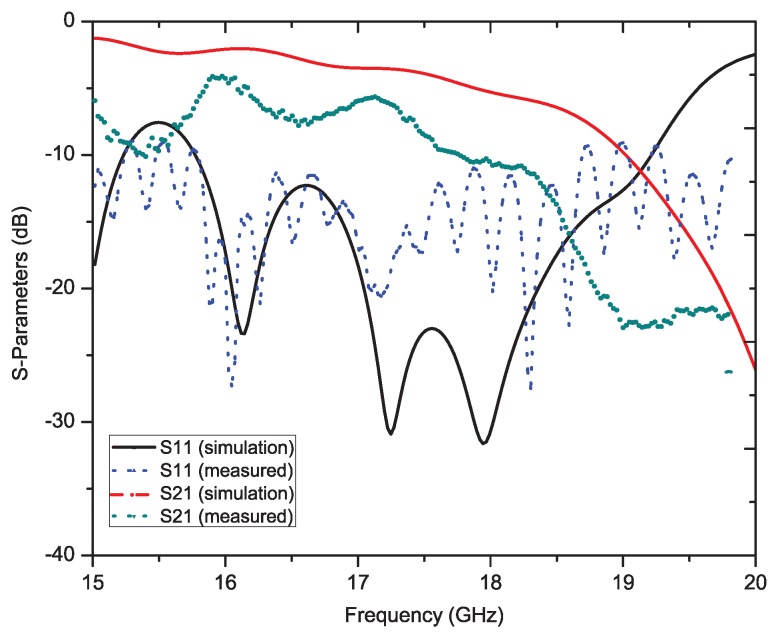
S-parameters of the proposed sine wave leaky wave antenna.

Figure 17 depicts the simulated and measured S-parameters (S11 and S22) for the proposed antenna design. For simulating the design, the transient solver in CST Studio Suite has been used. It can be clearly seen from Figure 17 that the antenna achieved a broadband impedance matching across the Ku-band. The maximum return loss was around 30 dB. S21 had some discrepancies with the measured results due to some soldering or handling-induced errors. The antenna has been fabricated on an extremely thin substrate. Still, in both cases, the values were good enough to achieve an efficient antenna design. Moreover, the measurement results were much better than the simulated performance in this case. The S21 parameter varied from −7 dB–−15 dB across the whole frequency band.

**Figure 18:**
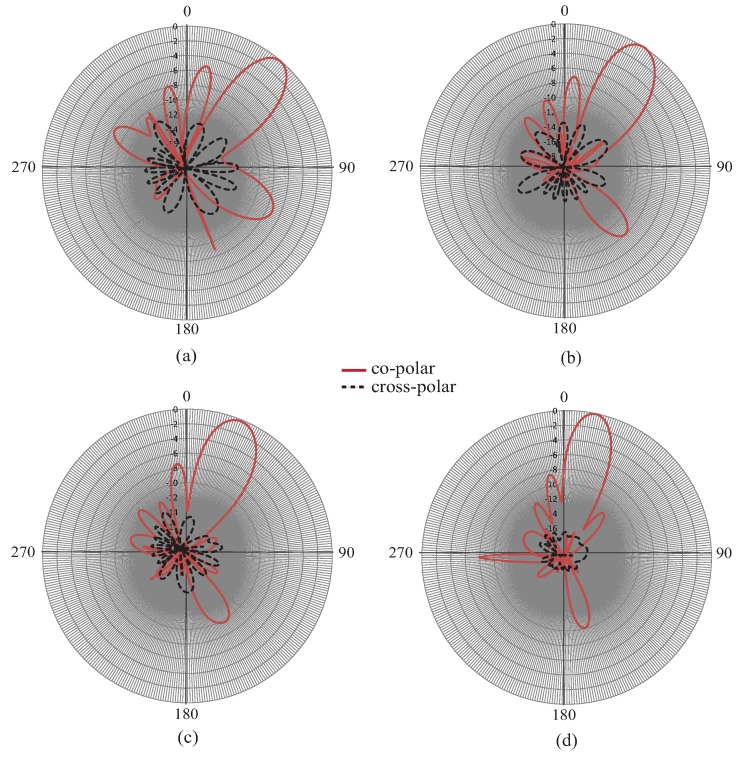
Radiation patterns with scanning at different frequencies: (**a**) 15.5 GHz, (**b**) 16.5 GHz, (**c**) 17.5 GHz, (**d**) 18.5 GHz.

**Figure 19:**
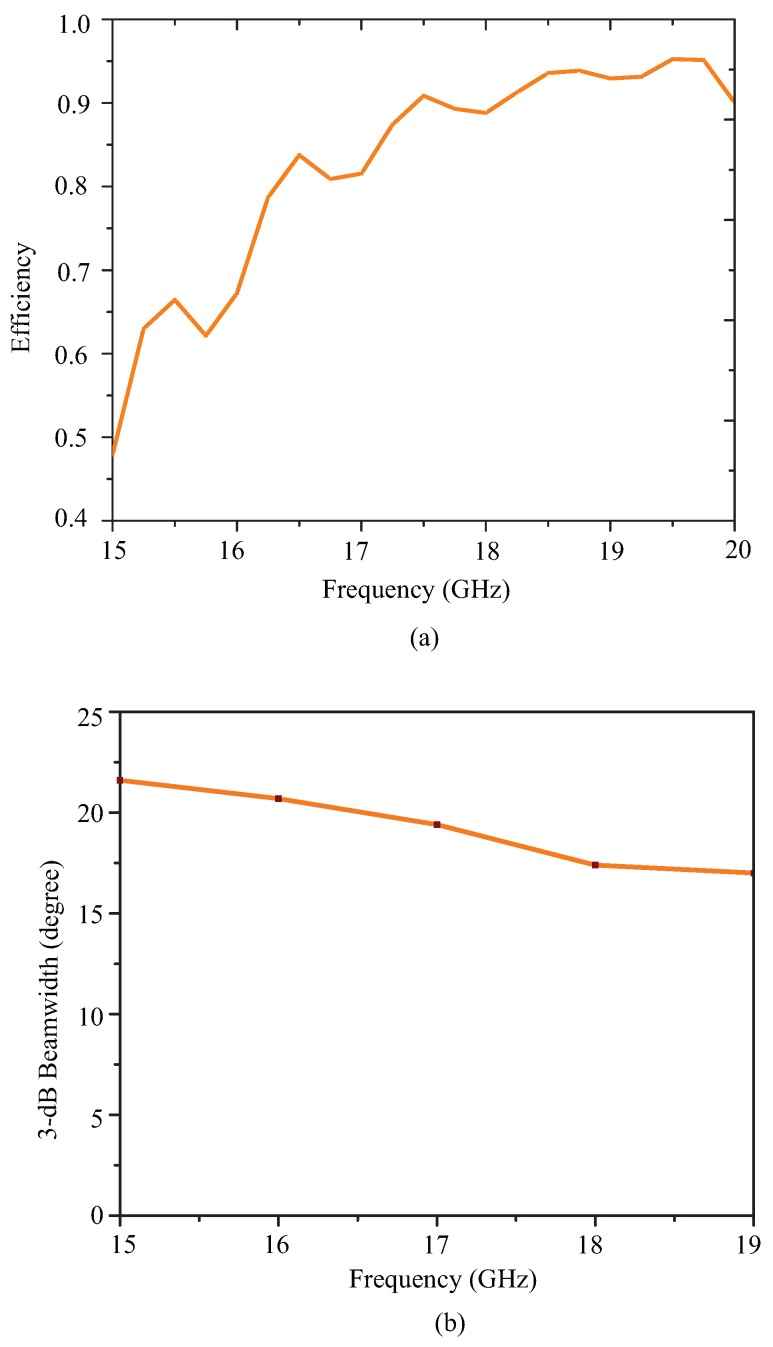
(**a**) Efficiency of the proposed sine wave leaky wave antenna and (**b**) 3-dB beamwidth variation with frequency.

**Figure 20:**
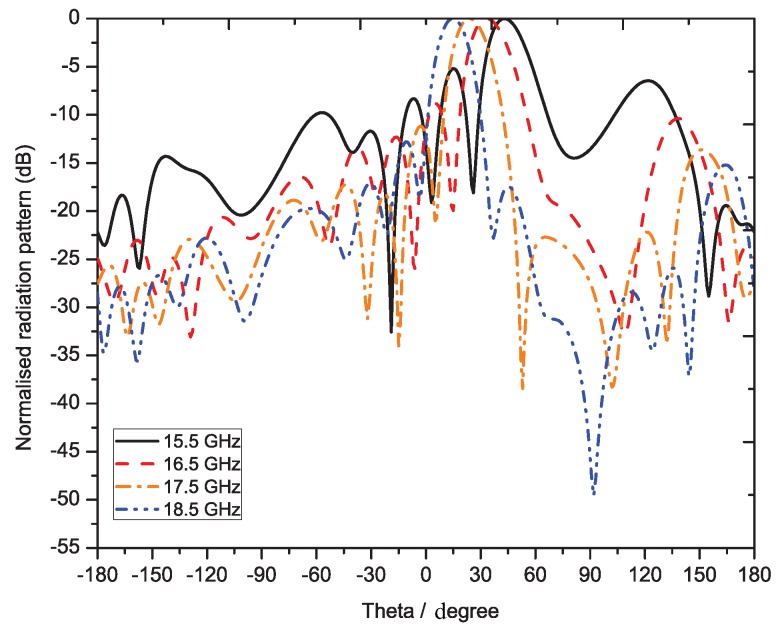
Measured radiation patterns at different frequencies of sine wave LWA.

Figure 18 shows the measured pattern of the proposed smooth edge sine wave leaky wave antenna. Figure 18 shows the power pattern at frequencies 15.0 GHz, 15.5 GHz, 16.0 GHz, 16.5 GHz, 17.0 GHz, 17.5 GHz, and 18.0 GHz. The antenna obviously exhibited a large scanning range of almost 50 degrees from 15 GHz–18 GHz. This type of right-hand scanning was observed as a result of the fast wave modes. Figure 19a,b shows the efficiency and 3-dB beamwidth graphs of the sine wave LWA as a function of frequency. The efficiency increased to 95 percent as the frequency increased from 15.0 GHz–18.0 GHz. However, the sidelobe levels at frequencies below 16 GHz were slightly higher as compared to those at other frequencies. At these frequencies (i.e., below 16 GHz), the S21 was slightly higher, but beyond this frequency, the efficiency continued to improve. In the desired operating frequency band (from 16-GHz onwards), the leakage radiation increased as the frequency went higher. Due to the frequency-dependent increase in leakage radiation along the transmission line, the gain also increased from lower frequencies to higher frequencies. At higher frequencies, the efficiency reached 90–95 percent, thus satisfying the criteria of a typical leaky wave antenna. Figure 19b shows the variation of the 3-dB beamwidth with the frequency. Figure 20 shows the measured normalized radiation patterns at different frequencies in the dB scale. Across the whole frequency band, the gain varied from 11 dBi–14 dBi. Consistent with the efficiency plot, the gain trended upward as the frequency increased. The minimum cross-polar levels obtained were around −20 dB in the operating frequency band.

## 5. Conclusions

Transmission lines periodically loaded with bends of different sharpness have been proposed in this paper for the realization of leaky wave antennas. The results of our simulation, mathematical modeling, as well as experimentation suggest that transmission lines periodically modulated with sharp bends exhibit both left-hand and right-hand scanning behaviors. On the other hand, transmission lines periodically loaded with smooth bends exhibit right-hand scanning behavior only. The proposed methodology has been successfully used to realize two leaky antennas, with the one modulated with sharp bends for composite right-hand/left-hand (CRLH) scanning and the other one modulated with smooth bends for right-hand scanning. The peak efficiencies for both fabricated leaky antennas reached 90 percent along with wide band impedance matching and high gain in the operating frequency bands of our choice. The proposed technique has opened up fundamentally new methodologies for leaky wave designs for applications including sensor and frequency scanning applications.

## Abbreviations

The following abbreviations are used in this manuscript:

CRLH	Composite right-hand/left-hand
LWA	Leaky wave antennas
SIW	Substrate integrated waveguide
